# Pseudotyping retrovirus like particles vaccine candidates with Hepatitis C virus envelope protein E2 requires the cellular expression of CD81

**DOI:** 10.1186/s13568-019-0741-5

**Published:** 2019-02-07

**Authors:** Hugo R. Soares, Rute Castro, Hélio A. Tomás, Manuel J. T. Carrondo, Paula M. Alves, Ana S. Coroadinha

**Affiliations:** 1 iBET, Instituto de Biologia Experimental e Tecnológica, Apartado 12, 2780-901 Oeiras, Portugal; 2 Instituto de Tecnologia Química e Biológica António Xavier, Universidade Nova de Lisboa, Av. da República, 2780-157 Oeiras, Portugal; 3 Departamento de Química, Faculdade de Ciências e Tecnologia, Universidade Nova de Lisboa, Monte da Caparica, Portugal

**Keywords:** HCV, CD81, Retrovirus, E2, E1, Transport

## Abstract

Hepatitis C virus (HCV) infects 3% of world population being responsible for nearly half a million deaths annually urging the need for a prophylactic vaccine. Retrovirus like particles are commonly used scaffolds for antigens presentation being the core of diverse vaccine candidates. The immunogenicity of host proteins naturally incorporated in retrovirus was hypothesized to impact the performance of retrovirus based vaccines. In this work, the capacity of engineered retrovirus like particles devoided of host protein CD81 to display HCV envelope antigens was compared to non-engineered particles. A persistent inability of CD81 negative VLPs to incorporate HCV E2 protein as a result from the inefficient transport of HCV E2 to the plasma membrane, was observed. This work enabled the identification of a CD81-mediated transport of HCV E2 while stressing the importance of host proteins for the development of recombinant vaccines.

**Electronic supplementary material:**

The online version of this article (10.1186/s13568-019-0741-5) contains supplementary material, which is available to authorized users.

## Introduction

With no licensed prophylactic vaccine, Hepatitis C virus (HCV) is considered one of the most important human pathogens of the XXI century, infecting approximately 80 million people worldwide (Baumert et al. 2014; Gower et al. 2014). The high genetic diversity of HCV genotypes, the extended capacity of HCV to circumvent immune defenses and the absence of suitable animal models to evaluate vaccine efficacy are major challenges for HCV vaccine development (Bellier and Klatzmann 2013). Additionally, the risk of an asymptomatic HCV infection associated to vaccine reversion limits the use of classical inactivated and live-attenuated vaccine candidates.

As a result, HCV vaccine candidates undergoing pre-clinical and clinical evaluation use non-conventional antigens such as vectored vaccines, recombinant protein cocktails and virus-like particles (VLPs) (Garrone et al. 2011; Huret et al. 2013). The latter have shown to be potent vaccine antigens as a result from their particulate nature even in the absence of adjuvants (Bellier et al. 2006, 2009; Gogesch et al. 2018). Virus-like particles based in Murine Leukemia virus (MLV) are the core of diverse vaccine candidates against pathogens such as Cytomegalovirus, West Nile fever virus, Hepatitis C virus and human immunodeficiency virus due to their capacity to incorporate high amounts of foreign membrane proteins (Bellier et al. 2006, 2009; Desjardins et al. 2009; Kirchmeier et al. 2014; Pitoiset et al. 2017). Murine Leukemia virus acquires its lipid envelope by extracting a fragment of the plasma membrane during budding stage which results in the incorporation of host membrane proteins in fully assembled particles (Segura et al. 2008; Soares et al. 2016). We have previously shown that host proteins incorporated in MLV-based VLPs, or retroVLPs, such as the tetraspanins CD81, CD63 and CD9, trigger the production of antibodies in mice thus conferring retroVLPs their basal immunogenity (Soares et al. 2016).

Similar to retrovirus, host proteins are known to associate with HCV virions (Lussignol et al. 2016). Even though the immunogenic profile of these host proteins is unknown, chronic HCV infections often result in the production of autoreactive antibodies (Roughan et al. 2012). Here we hypothesize the use of engineered retroVLPs with reduced CD81-specific immunogenicity to study the role of host proteins in HCV immunity and to support the development of engineered HCV vaccine candidates.

In this work CD81 positive and CD81 negative cells were used to produce retroVLPs pseudotyped with HCV envelope proteins. An inefficient transport of HCV E2 to the plasma membrane of CD81 silenced cells however hampered HCV pseudotyping of retroVLPs in these cells. The inefficient transport of HCV E2 was further confirmed in cells producing HCV-like particles. Contrasting with the prevalent hypothesis arguing for an inefficient retention of viral proteins in the endoplasmic reticulum (ER) (Bartosch et al. 2003; Op De Beeck et al. 2004) our results suggest the existence of a CD81-mediated transport of HCV E2 to the plasma membrane. Our results also show that the transport of HCV E1 is independent from HCV E2 and CD81 thus contributing for the development of E1-only vaccine candidates.

## Materials and methods

### Cell lines and culture media

293rVLP and 293rVLP-shCD81 cell lines derive from HEK 293 (ATCC CRL-1573) by stable expression of MLV gag-pro-pol gene for continuous production of recombinant retrovirus products (Rodrigues et al. 2011). 293rVLP-HCVpp and 293rVLP-HCVpp shCD81 (BEE) cells derived from the previous cells by stable transfection of pEPX145-71 plasmid expressing HCV E1 and E2 genes (Garrone et al. 2011) and pMonoZeoMCS, a plasmid containing the zeocin resistance gene in a ratio 1:20 (m/m), using 1 µg P.E.I. to 1.5 µg of DNA. 293rVLP-HCVpp shCD81 (AEE) cells derive from 293rVLP-HCVpp upon silencing endogenous CD81 expression. To establish HCV-LP producer cells, HuH-7 (JCRB 0403) and HEK 293 (ATCC CRL-1573) were used. All cells were maintained in Dulbecco’s modified Eagle’s medium (DMEM) (Gibco, Carlsbad, CA, USA) supplemented with 10% (v/v) fetal bovine serum (FBS) (Gibco) at 37 °C inside an incubator with a humidified atmosphere of 5% CO_2_ in air. A summary table with all cell lines used and developed is available as Additional file 1: Table S1.

### Cell transduction and selection

For the establishment of de novo CD81 silenced cell populations 293rVLP-HCVpp, 293-HCV_Core-NS2_ and HuH7-HCV_Core-NS2_ cells were seeded in six-well plates at 5 × 10^4^ cells/cm^2^; 24 h later, culture medium was replaced by 600 µL of lentiviral supernatant containing shRNA#10, (Rodrigues et al. 2011) targeting CD81 supplemented with 8 mg/mL of Polybrene (Sigma-Aldrich). After 4 h incubation at 37 °C, 1.5 mL of DMEM 10% FBS (v/v) was added. 48 h post-infection, cells were expanded and cultured for 21 days in DMEM containing puromycin (1.5 mg/mL). For further enhancement of CD81 down-regulation, initial shCD81#10 cell populations were subsequently infected with shCD81 #9 and #12, as described previously (Rodrigues et al. 2011), followed by 14 days selection with 3 mg/mL puromycin, establishing the final shCD81 cell population. The development of 293-HCV_Core-NS2_, HuH-7-HCV_Core-NS2_ and corresponding shCD81 derived cells is summarized in Additional file 1: Figures S4 and S5 schematic representations.

### Western Blotting

Cell lysates were prepared by adding 100 µL of M-PER extraction buffer (Thermo Scientific, Waltham, MA, USA) per 1 × 10^6^ cells. The total protein concentration in cell lysates or purified virus samples was determined using Pierce™ BCA Protein Assay Kit (Thermo Scientific). 15 µg of protein extracts or purified virus like particles were separated in a 4–12% (w/v) acrylamide NuPAGE gradient pre-cast gel (LifeTechnologies). Samples were resolved for 40 min at a constant voltage of 180 V and transferred into a PVDF membrane (Merck, Billerica, Massachusetts, EUA) using a Trans-Blot^®^ Turbo™ Transfer System (BioRad, California, USA). After transferring, PVDF membranes were blocked with 4% (w/v) skimmed milk (Merck) and incubated with the respective primary antibody: rabbit anti-CD81 (Sigma-Aldrich), mouse anti-β-Tubulin antibody (SantaCruz, CA, USA), mouse anti-HCV E1 (Acris Antibodies, Herford, Germany), mouse anti-HCV E2 (Austral Biologicals), mouse anti-HCV Core (SantaCruz) or the anti-MLV p30 monoclonal antibody produced by the hybridoma R187 (ATCC). Detection was performed with the corresponding anti-mouse or anti-rabbit secondary antibody conjugated to Horseradish peroxidase and developed using the ECL Detection Reagent (GE Healthcare, Chicago, IL USA).

### Flow cytometry

To determine the presence of CD81 and HCV E2 on the cell surface 100 µL of a 1 × 10^6^ cells/mL suspension were incubated with rabbit anti-CD81 (Sigma-Aldrich) and anti-HCV E2 (Austral Biologicals) monoclonal antibodies for 1 h, shaking at 4 °C. After extensive washing with PBS 2% (v/v) FBS, cells were incubated with Goat anti-Rabbit A594 (Thermo Scientific) and Goat anti-mouse A488 (Thermo Scientific) conjugated antibodies for an additional hour at 4 °C. At the end, cells were washed 3× with PBS 2% (v/v) FBS and the percentage of fluorescence cells was determined by flow cytometry using CYFlow space flow cytometer (Partec, Münster, Germany). Data acquisition was performed with FlowMax (Partec) software and data analysis with FlowJo v10 (FlowJo, LLC, Ashland, Ore USA).

## Results

### CD81 silencing impairs HCV E2 but not HCV E1 transport to the plasma membrane

**Fig. 1:**
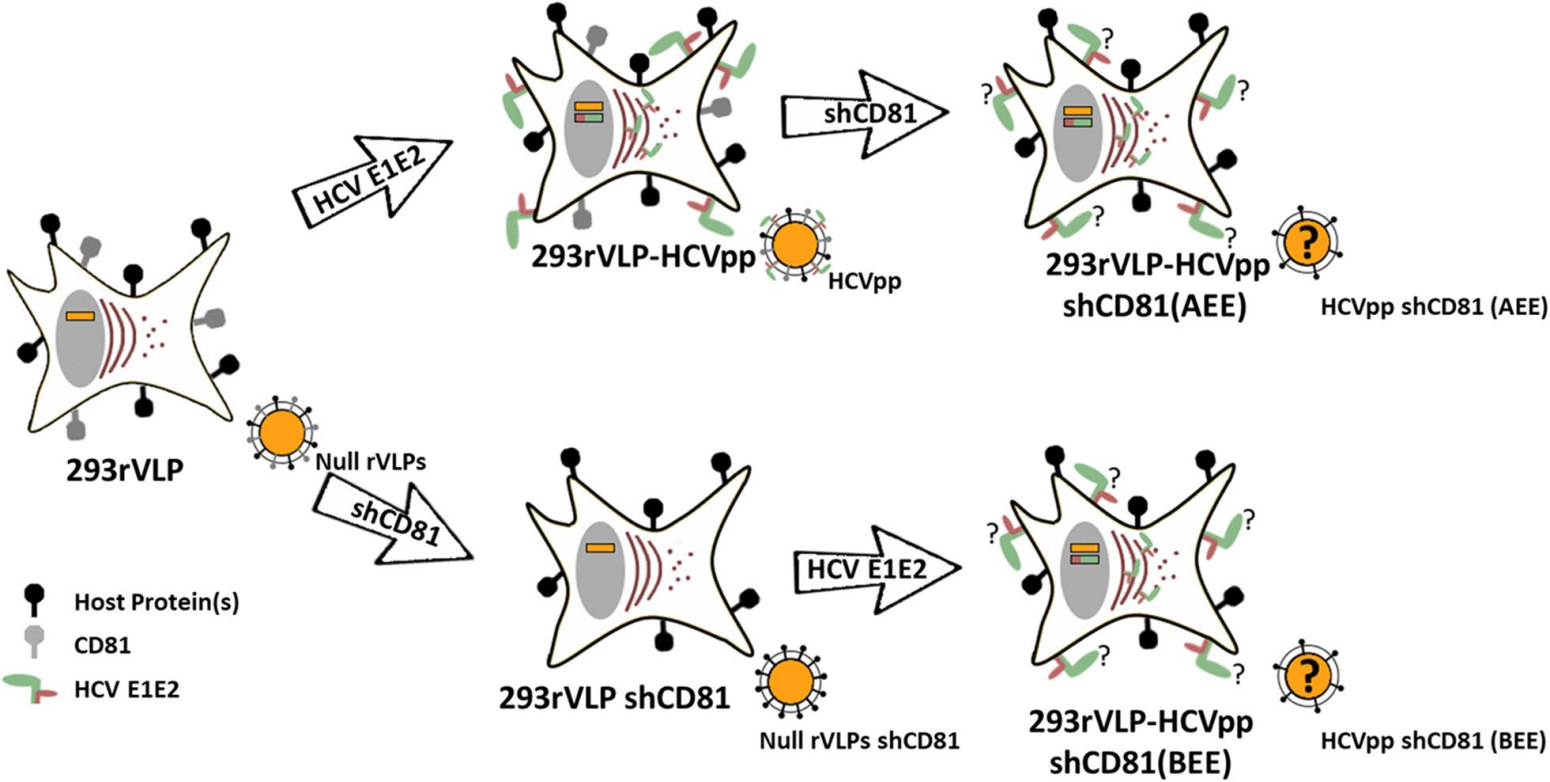
Schematic representation of cell line development. 293rVLP cells producing non pseudotyped retrovirus like particles (Null VLPs) were transfected with HCV envelope E1 (green) and E2 (brown) proteins coding plasmid and produced HCV pseudoparticles (HCVpp), afterwards these cells were silenced for endogenous CD81 generating 293rVLP-HCVpp shCD81 (AEE—After Envelope Expression) (upper panel). In parallel, 293rVLP were silenced for endogenous CD81 expression to produce non-pseudotyped retrovirus like particles deprived from CD81, these cells were then transfected with a plasmid coding HCV envelope proteins generating 293rVLP-HCVpp shCD81 (BEE—Before Envelope Expression) (lower panel)

**Fig. 2:**
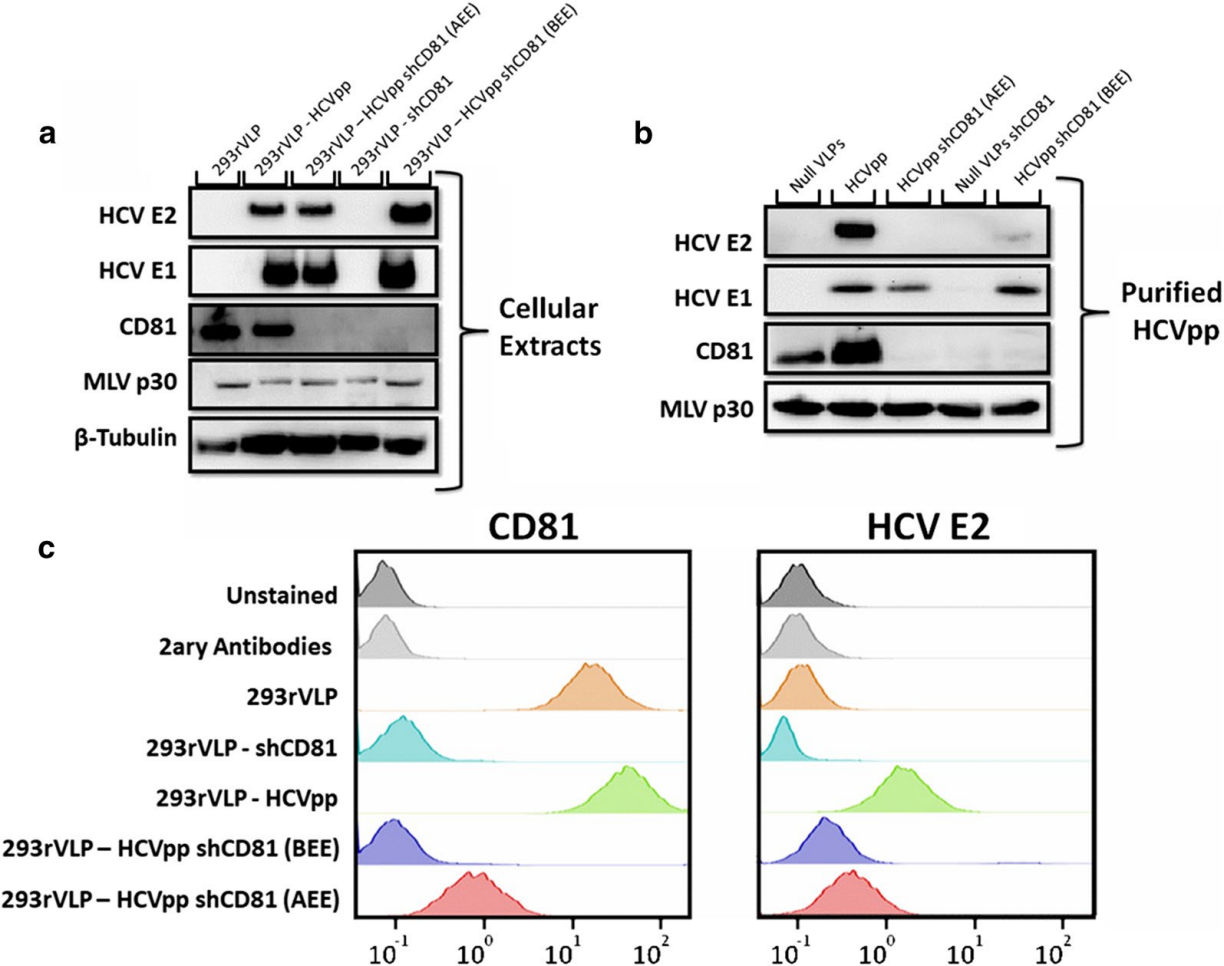
Cellular expression of and incorporation of HCV envelope proteins in chimeric HCVpp. **a** Cellular expression of MLV p30, CD81, HCV E1 and HCV E2 determined by western blotting in 293rVLP, 293rVLP-HCVpp, 293rVLP-HCVpp (AEE), 293rVLP-shCD81 cells and 293rVLP-HCVpp-shCD81 (BEE); **b** incorporation of CD81, HCV E1 and HCV E2 membrane proteins in null retrovirus particles or HCVpp produced in 293rVLP, 293rVLP-HCVpp, 293rVLP-HCVpp (AEE), 293rVLP-shCD81 cells and 293rVLP-HCVpp (BEE) cells determined by western blotting of purified particles; **c** surface displayed CD81 and HCV E2 proteins 293rVLP, 293rVLP-HCVpp, 293rVLP-HCVpp (AEE), 293rVLP-shCD81 cells and 293rVLP-HCVpp (BEE) cells determined by flow cytometry

To study the influence of viral CD81 in HCV immunity and vaccine performance, endogenous CD81 expression was silenced in retroVLPs producing cells before and after inducing the heterologous expression of HCV envelope proteins (Fig. 1; Additional file 1: Figures S1, S2). These cells were named 293rVLP-HCVpp shCD81 (AEE) and 293rVLP-HCVpp shCD81 (BEE) cells, as a reference to CD81 silencing occurring After Envelope Expression or Before Envelope Expression, respectively. HCVpp refers to HCV pseudoparticles which are retroviral particles pseudotyped with HCV envelope proteins. The expression of CD81, HCV envelope proteins and MLV structural protein p30 was evaluated in all engineered cell lines and derived particles. As shown in Fig. 2a and Additional file 1: Figure S3, the cellular expression of all viral proteins in CD81-positive and in CD81-negative cells is similar. On the other hand the analysis of envelope proteins incorporated on viral particles (Fig. 2b) revealed a reduced incorporation of HCV E2 in HCVpp produced by CD81-silenced cell lines while HCV E1 incorporation levels were maintained. To evaluate the transport of HCV E2 to retroVLPs budding site at the plasma membrane, the display of HCV E2 on the cell surface was determined by flow cytometry. As shown in Fig. 2c, HCV E2 is consistently lower in CD81-silenced cells in comparison to non-silenced cells, thus explaining the lower incorporation of HCV E2 on budding HCVpp.

### CD81-dependent transport of HCV E2 in HCV-core expressing cells

**Fig. 3:**
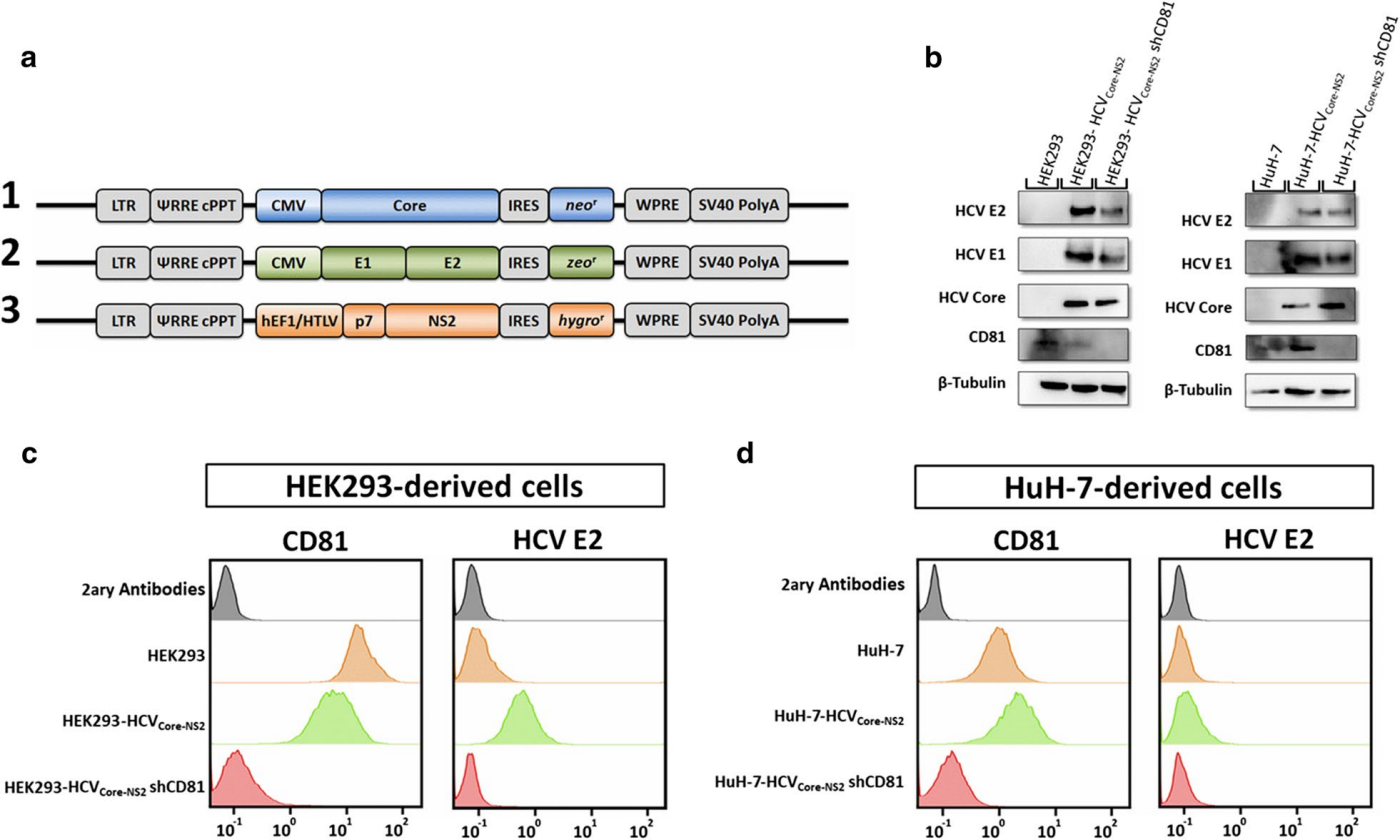
Validation of CD81-dependent transport of HCV E2 in HCV core expressing cells. **a** Schematic representation of the expression plasmids containing lentiviral vectors transgene sequences inducing the cellular expression of HCV Core, E1E2 and p7NS2 ORFs; **b** cellular expression of MLV p30, CD81, HCV E1 and HCV E2 determined by western blotting in HEK293, HEK293-HCV_Core-NS2_, HEK293-HCV_Core-NS2_ shCD81, HuH-7, HuH7-HCV_Core-NS2_ and HuH7-HCV_Core-NS2_ shCD81. **c** Surface displayed CD81 and HCV E2 proteins in (**c**) HEK293, HEK293-HCV_Core-NS2_, HEK293-HCV_Core-NS2_ shCD81 and **d** HuH-7, HuH7-HCV_Core-NS2_ and HuH7-HCV_Core-NS2_ shCD81

To investigate the existence of a CD81-dependent transport of HCV E2 in cells producing HCV particles, endogenous CD81 expression was silenced in HEK293 and HuH-7 cells expressing HCV viral proteins (Core, E1, E2, p7 and NS2) (Fig. 3a and Additional file 1: Figure S4). HCV proteins were overexpressed in three independent open reading frames originating HEK293_Core-NS2_ and HuH-7_Core-NS2_ cells (Fig. 3a and Additional file 1: Figure S4). The silencing of endogenous CD81 expression after the stable expression of HCV proteins (Fig. 3a and Additional file 1: Figure S4) did not alter the cellular expression levels of viral proteins (Fig. 3b). Nonetheless, a reduction in cell surface exposed HCV E2 is observed in both HEK293_Core-NS2_ shCD81 and HuH-7_Core-NS2_ shCD81 (Fig. 3c, d) similar to HCVpp producing cells. Furthermore, CD81 silencing did not affect the intracellular assembly of HCV-LP occurring at the endoplasmic reticulum (Additional file 1: Figure S5).

## Discussion

The emergence of virus-like particles (VLPs) as vaccine scaffolds enabled the development of vaccine candidates for conditions which other technologies have failed. MLV based particles were shown to efficiently incorporate HCV envelope antigens from different genotypes and to generate antibodies able to cross-neutralize all tested genotypes thus generating protective immunity (Garrone et al. 2011; Huret et al. 2013; Urbanowicz et al. 2015).

We have previously shown that host proteins present in MLV particles, such as CD81, are responsible for retroVLPs basal immunogenity (Soares et al. 2016). Since CD81 is a crucial host-factor for Hepatitis C virus, the impact of CD81 incorporation and the generation of anti-CD81 antibodies in the performance of retroVLPs based vaccines was questioned.

In 2004, Masciopinto et al. (2004) showed that hCD81 expression in CHO-cells promoted the transport of HCV envelope proteins to the plasma membrane and consequently their incorporation in exosomes. Regardless, the idea of a non-regulated process in which a small fraction of viral proteins escaping the ER retention machinery are incorporated in the plasma membrane prevails as the mechanism responsible for the incorporation of HCV envelope proteins in chimeric viral particles (Bartosch et al. 2003; Op De Beeck et al. 2004). Here we validate the transport of HCV E2 envelope protein to the plasma membrane as a regulated process dependent from cellular CD81 expression.

To further validate our observations, CD81 negative HCVpp were generated independently and produced in a clonal cell line or in a heterogeneous cell population. To enable a direct comparison between cell lines generated independently, single cell clones with similar expression of viral proteins were isolated and named 293rVLP-HCVpp and 293rVLP-HCVpp shCD81 (BEE) (Additional file 1: Figures S1 and S2). To minimize the potential amplification of unwanted characteristic underrepresented in the initial cell population during clonal selection, CD81 was de novo silenced in 293rVLP-HCVpp generating 293rVLP-HCVpp shCD81 (AEE) which was maintained as an heterogeneous cell population. A schematic representation of cell line development is shown in Fig. 1 and summarized in Additional file 1: Table S1.

The evaluation of protein expression levels in all generated cell lines indicates similar expression of HCV envelope proteins, similar silencing of CD81 and maintenance of MLV p30 expression levels (Fig. 2a). Notwithstanding similar protein expression, the levels of HCV E2 envelope protein incorporated by secreted viral particles and exposed at cell surface was consistently lower in CD81-silenced cells suggesting an inefficient transport of HCV E2 to the plasma membrane (Fig. 2b, c). The coherent reduction of surface exposed HCV E2 in HEK293 and HuH-7 cells producing HCV-LP after CD81 silencing further validates the existence of an in *cis* interaction between intracellular CD81 and HCV E2 responsible for HCV E2 transport to the membrane.

In contrast, CD81 silencing had no impact in the incorporation of HCV E1 on HCVpp suggesting independent transport mechanisms. The independent transport of HCV E1 grants the possibility to generate viral particles pseudotyped exclusively with HCV E1 using natural HCV sequences through cell line engineering. A chimeric HCV E1 protein enabling E1-only pseudotyping of retroVLPs, was shown to enhancing the protective potential of a vaccine candidate by increase the prevalence of rare anti-HCV E1 antibodies when administered in combination with fully pseudotyped particles in a prime-boost vaccination strategy (Garrone et al. 2011; Huret et al. 2013).

Overall, this study provides evidences that HCV envelope protein E2 transport is regulated by its intracellular association with cellular CD81. The validation of a CD81-regulated transport of HCV E2 to the plasma membrane can further contribute to elucidate understudied aspects of HCV biology such as the biogenesis of infectious HCV-genome containing exosomes present in patients’ serum. In addition, this work grants the possibility to develop HCV E1-only particles using non-engineered viral envelopes and highlights the importance of cellular host proteins for the production of functional vaccine candidates.

## Additional file

**Additional file 1.** Additional Materials and Methods, Results, Tables and References.
