# Supplementary material for: Pseudotyping retrovirus like particles vaccine candidates with Hepatitis C virus envelope protein E2 requires the cellular expression of CD81
Source: AMB Express. 2019 Feb 7;9:22. doi: 10.1186/s13568-019-0741-5 (PMC6367494; doi:10.1186/s13568-019-0741-5)
Supplement: Supplementary file 1 — Additional file 1. Additional Materials and Methods, Results, Tables and References. [file 13568_2019_741_MOESM1_ESM.docx]

**AMB Express Additional Information**

**Pseudotyping retrovirus like particles vaccine candidates with Hepatitis C virus envelope protein E2 requires the cellular expression of CD81**

Hugo R. Soares^1,2^, Rute Castro^1,2^, Hélio A. Tomás^1,2^, Manuel J. T. Carrondo^1,3^, Paula M. Alves^1,2^, Ana S. Coroadinha^1,2^#

^1^iBET, Instituto de Biologia Experimental e Tecnológica, Apartado 12, 2780‑901 Oeiras, Portugal

^2^Instituto de Tecnologia Química e Biológica António Xavier, Universidade Nova de Lisboa, Av. da República, 2780-157 Oeiras, Portugal

^3^Departamento de Química, Faculdade de Ciências e Tecnologia, Universidade Nova de Lisboa, Monte da Caparica, Portugal

**#Correspondence:**

Dr. Ana Sofia Coroadinha

iBET – Instituto de Biologia Experimental e Tecnológica, Apartado 12, 2781-901 Oeiras, Portugal

Telephone: +351 21 4469457

e-mail: avalente@ibet.pt

**Additional Materials and Methods**

**Plasmids**

The plasmids used for the generation of lentiviral vectors, pMDLg/pRRE, pRSV-REV, pMD2.G and pRRLIN.hPGK.GFP.wPRE were kindly provided by Prof. Didier Trono trough Addgene (Cambridge, MA). pMDLg/pRRE drives the expression of HIV-1 *gag/pol* gene, pRSV-REV encodes the auxiliary protein REV, pMD2.G encodes VSV-G envelope protein and pRRLIN.hPGK.GFP.wPRE encodes the lentiviral transgene with a GFP reporter gene. The plasmids used for de novo silencing of CD81 are based on pLKO.1-puro and were described previously (Rodrigues et al., 2011). Expression of HCV envelope proteins E1 and E2 from strain H77 in 293rVLP and 293rVLP-shCD81 was accomplished by co-transfecting pEPX145-71 plasmid (Garrone et al., 2011) and pMonoZeoMCS (InvivoGen, San Diego, U.S.A.).

**Lentiviral vectors production**

To produce third generation lentiviral vectors, HEK 293T cells were seeded at a concentration of 5x10^4^ cell / cm^2^ in 25 cm^2^ tissue culture flasks (Sarstedt, Nümbrecht Germany), 24 hours later cells were transfected using linear 25 kDa polyethyleneimine (PEI; Polysciences Inc, Germany) in a proportion of 1:1.5 (DNA : PEI), with the respective plasmids. The amount of each viral component per 1x10^6^ cells was as follows: 2.5 µg of the respective vector transgene plasmid; 1 µg of pMDLg/pRRE; 0.25 µg of pRSV-REV and 0.9 µg of pMD2.G. Transfection procedure for a 25 cm^2^ tissue culture flask: 0.3 mL of DMEM with PEI were added to 0.3 mL of DMEM with DNA and mixed thoroughly. After 15 min of incubation at room temperature the mixture of PEI-DNA was added to the cells. After 24 hours, the medium was replaced by fresh medium. 24 hours later the supernatant was harvested, filtered at 0.45 µm and cryopreserved frozen at -85ºC.

**Immunofluorescence**

HEK293 and HEK293 derived cells were seeded onto poly-D-lysine pre-treated coverslips at a concentration of 1x10^5^ cell / cm^2^ and cultured overnight. On the next day, cells were fixed using 4% (w/v) PFA (Sigma-Aldrich, St. Louis, MO, U.S.A.) during 20 minutes at room temperature and permeabilized with PBS with 0.1 % (v / v) Triton X-100 for an additional 10 minutes. Permeabilized cells were blocked using PBS 0.2 % (v / v) Fish Skin Gelatin (Sigma-Aldrich) during 30 minutes at room temperature. Incubation with primary antibodies was performed overnight at 4 °C using anti-CD81 (Sigma-Aldrich), anti-HCV E2 (Austral Biologicals, CA, U.S.A.) or anti-MLVp30 (Hybridoma R187, ATCC CRL-1912) antibodies. All washes were performed at room temperature with PBS 0.05 % (v / v) Tween20. ProLong® Gold Antifade Reagent with DAPI (LifeTechnologies, Carlsbad, CA, U.S.A.) was used as mounting media. Samples were observed using Leica DMI6000 inverted microscope (Leica, Wetzlar, Germany).

**Virus like particles production and purification**

Virus like particles production and purification was performed as described before (Rodrigues et al., 2011; Soares et al., 2016). Briefly, cells were seeded in 225 cm2 tissue-culture flasks (Corning Life Sciences, Tewksbury, MA U.S.A.) and cultured until 80 % of cellular confluence. For retroVLP production, culture medium was then replaced with new culture medium which was harvested after 24 hours production. Cell culture medium was filtered for clarification and purified by a two-step ultracentrifugation, first at 60 000 x g for 2 hours, and a second one at 120 000 x g in a 20 % (w / v) sucrose cushion for 2 additional hours, both centrifugations were performed at 4º C.

**Additional Results**

**Development of HCVpp producer cell-lines**

To study the influence of CD81 in HCV envelope protein trafficking, 293rVLP and 293rVLP-shCD81 cells which are continuously secreting empty, non-enveloped, murine leukemia virus (MLV) based particles were stably transfected with a plasmid encoding HCV E1 and E2 to produce HCVpp and selected with zeocin. The cell populations obtained were tested for HCV E1 and E2 expression by Western blotting analysis (Fig S1). These populations were cloned into single cells by limiting dilution aiming to expand a single cell into a homogeneous cell population. A maximum of 20 individual clones were isolated from 293rVLP and 293rVLP-shCD81 parental cells and numbered 1-29 or 30 to 49, respectively, and cryopreserved. A limited set of clones, based in their cell growing profile were screened for HCV E1 expression by western blotting (Fig. S2). Cell clones #9 and #38 were selected based in their similar expression of HCV E1 protein and re-named 293rVLP-HCVpp and 293rVLP-HCVpp shCD81 (BEE), respectively. The overall HCV E2 expression levels, subcellular distribution and of co-localization with MLV p30 and CD81 was assessed by fluorescence microscopy (Fig. S3). Images suggest conservation of HCV E2 and MLV p30 proteins expression and distribution patterns in CD81 silenced cells.

**Development of 293rVLP-HCVpp shCD81 (AEE)**

To study directly the effect of CD81 silencing in HCV E2 transport and minimize the effect of clone-specific characteristics amplified during clonal cell expansion, cellular CD81 was silenced in cells producing fully pseudotyped HCVpp, 293rVLP-HCVpp. Here, surviving cells were maintained as an heterogeneous cell population and named 293rVLP-HCVpp shCD81(AEE).

**Additional Figures**

**Anti-HCV E2**

**Anti-HCV E1**

**Figure S1 –** Detection of HCV-E1 and HCV-E2 in transfected cells populations. (A) SDS-PAGE and (B and C) Western blotting profile of 293rVLP (1), 293rVLP transfected with pEPX141-71 (2), 293rVLP shCD81 (3) and the 293rVLP shCD81 transfected with pEPX141-71 (4).


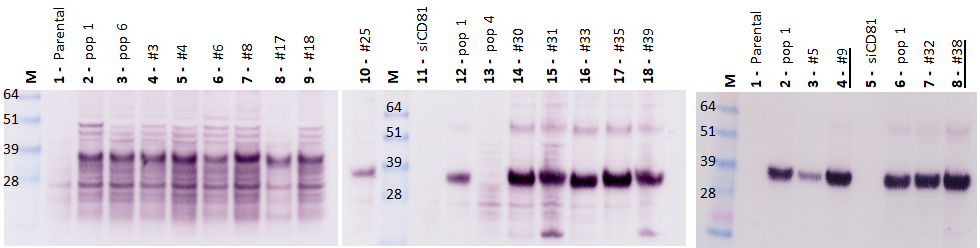


**Figure S2 –** Detection of HCV-E1 in cell extracts of two populations and individual cell clones. Western blotting profile of 293rVLP-HCVpp derived clones (#1 to #25), and 293rVLP-HCVpp shCD81 (BEE) derived clones (#30 to #39). Underlined clones #9 and #38 were selected for further studies.


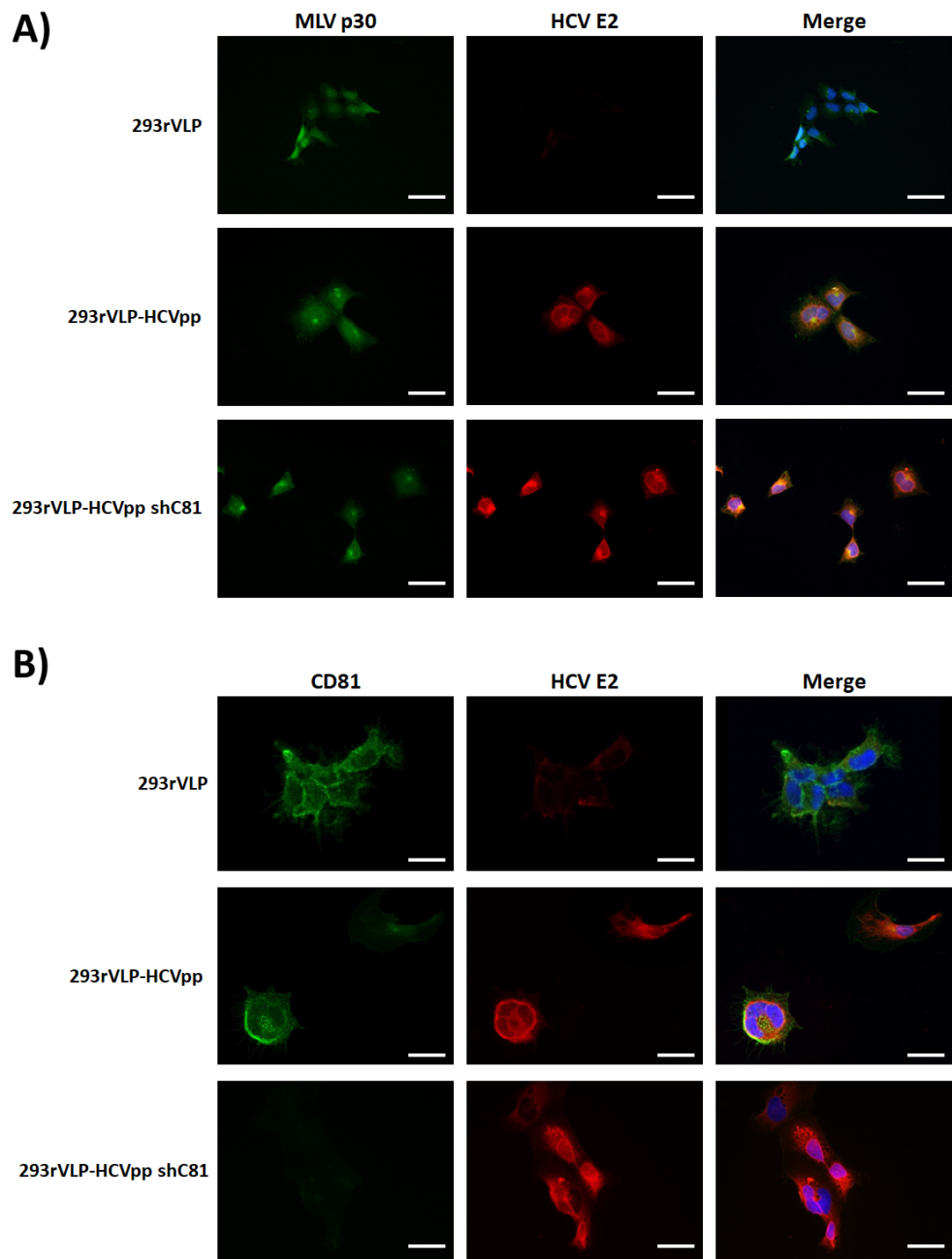


**Figure S3 –** Subcellular localization of HCV E2 and **(A)** MLV p30 and **(B)** CD81 in 293rVLP, 293rVLP-HCVpp and 293rVLP-HCVpp shCD81 (BEE) cells determined by fluorescence microscopy, cell nucleus are stained with DAPI (blue); white bars in the right bottom corner represent a 50 µm scale.


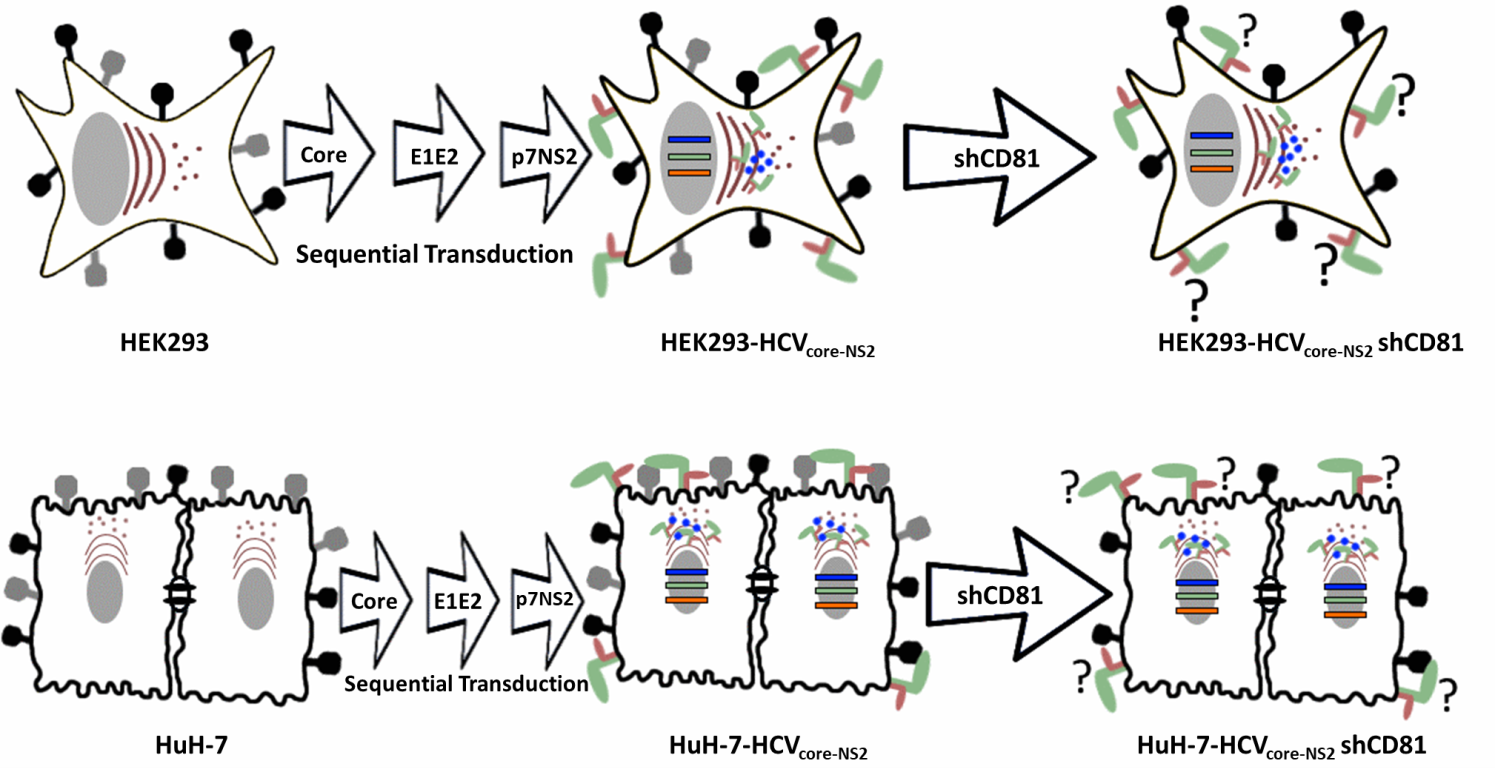


**Figure S4 –** Schematic representation of 293-HCV_Core-NS2_ shCD81 and HuH-7-HCV_Core-NS2_ shCD81 cells development. HEK293 and HuH-7 cells were transduced with lentiviral vectors encoding HCV assembly module proteins: Core, HCV envelopes E1 and E2 and HCV p7 and NS2 proteins aiming at HCV particles production, afterwards these cells were silenced for endogenous CD81. Blue dots inside the cell represent intracellularly entrapped HCV-LP.


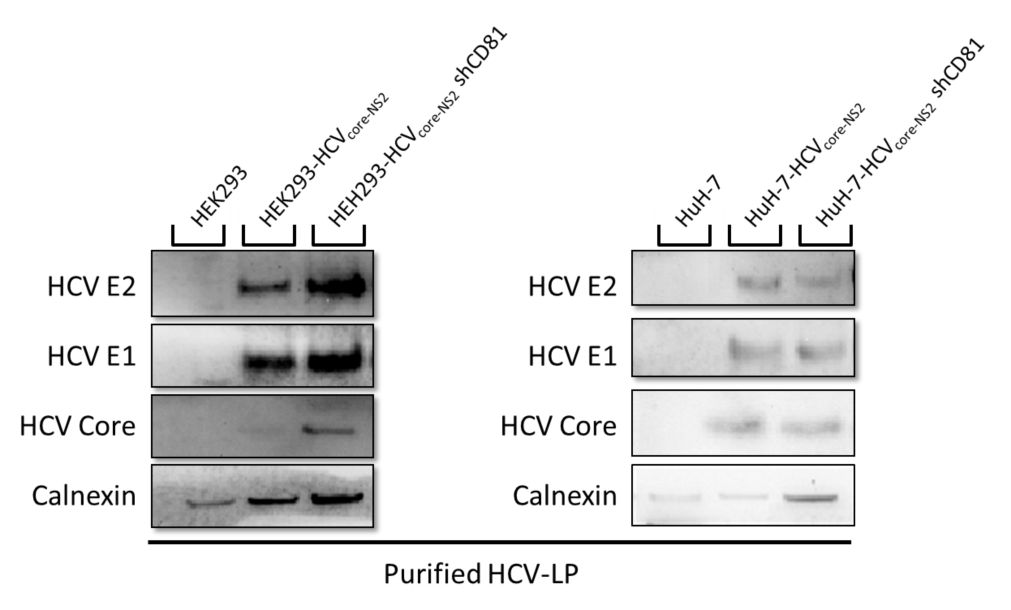


**Figure S5 –** Detection of HCV Core, E1 and E2 in intracellular HCV-LP formed after stable expression of HCV core, E1 and E2 in HEK293 and HuH-7 cells. HCV-LP were isolated from whole cell lysates through ultracentrifugation.

**Additional Table**

**Table S1.**

|  | **Cell Line** | **Clonality** | **Parental Cell Line** | **Overexpressed genes** | **Expression of CD81** | **Reference** |
| --- | --- | --- | --- | --- | --- | --- |
| **1** | 293rVLP | Single cell Clone | HEK293 (6) | MLV p30 | Endogenous | (Rodrigues et al., 2011; Soares et al., 2016) |
| **2** | 293rVLP HCVpp | Single cell Clone | 293rVLP (1) | MLV p30 ; HCV E1E2 | Endogenous | Present study |
| **3** | 293rVLP shCD81 | Single cell Clone | 293rVLP (1) | MLV p30 | Silenced | (Rodrigues et al., 2011; Soares et al., 2016) |
| **4** | 293rVLP shCD81 HCVpp (BEE) | Single cell Clone | 293rVLP shCD81 (3) | MLV p30 ; HCV E1E2 | Silenced | Present study |
| **5** | 293rVLP shCD81 HCVpp (AEE) | Population | 293rVLP HCVpp (2) | MLV p30 ; HCV E1E2 | Silenced | Present study |
| **6** | HEK293 |  | ATCC | - | Endogenous |  |
| **7** | 293-HCVCore-NS2 | Population | HEK293 (6) | HCV Core, E1, E2, p7, NS2 | Endogenous | Present study |
| **8** | 293-HCVCore-NS2 shCD81 | Population | 292 HCVCore-NS2 (7) | HCV Core, E1, E2, p7, NS2 | Silenced | Present study |
| **9** | HuH-7 | Single cell Clone | JCRB | - | Endogenous | (Nakabayashi et al., 1982) |
| **10** | HuH-7-HCVCore-NS2 | Population | HuH-7 (9) | HCV Core, E1, E2, p7, NS2 | Endogenous | Present study |
| **11** | HuH-7-HCVCore-NS2 shCD81 | Population | HuH-7 HCVCore-NS2 (10) | HCV Core, E1, E2, p7, NS2 | Silenced | Present study |

**Additional References**

Garrone, P., Fluckiger, A.-C., Mangeot, P.E., Gauthier, E., Dupeyrot-Lacas, P., Mancip, J., Cangialosi, A., Du Chéné, I., LeGrand, R., Mangeot, I., Lavillette, D., Bellier, B., Cosset, F.-L., Tangy, F., Klatzmann, D., Dalba, C., 2011. A prime-boost strategy using virus-like particles pseudotyped for HCV proteins triggers broadly neutralizing antibodies in macaques. Sci. Transl. Med. 3, 94ra71. doi:10.1126/scitranslmed.3002330

Nakabayashi, H., Taketa, K., Miyano, K., Yamane, T., Sato, J., 1982. Growth of human hepatoma cells lines with differentiated functions in chemically defined medium. Cancer Res. 42, 3858–63.

Rodrigues, A.F., Guerreiro, M.R., Santiago, V.M., Dalba, C., Klatzmann, D., Alves, P.M., Carrondo, M.J.T., Coroadinha, A.S., 2011. Down-regulation of CD81 tetraspanin in human cells producing retroviral-based particles: tailoring vector composition. Biotechnol. Bioeng. 108, 2623–33. doi:10.1002/bit.23231

Soares, H.R., Castro, R., Tomás, H.A., Rodrigues, A.F., Gomes-Alves, P., Bellier, B., Klatzmann, D., Carrondo, M.J.T., Alves, P.M., Coroadinha, A.S., 2016. Tetraspanins displayed in retrovirus-derived virus-like particles and their immunogenicity. Vaccine 34, 1634–1641. doi:10.1016/j.vaccine.2015.12.015
